# The Pelagic Bacterium *Paraphotobacterium marinum* Has the Smallest Complete Genome Within the Family *Vibrionaceae*

**DOI:** 10.3389/fmicb.2017.01994

**Published:** 2017-10-11

**Authors:** Zhaobin Huang, Zhen Liu, Zongze Shao

**Affiliations:** ^1^Key Laboratory of Marine Genetic Resources, Third Institute of Oceanography, State of Oceanic Administration, Xiamen, China; ^2^Fujian Key Laboratory of Marine Genetic Resources, Xiamen, China; ^3^Fujian Collaborative Innovation Center of Marine Biological Resources, Xiamen, China; ^4^South China Sea Bio-Resource Exploitation and Utilization Collaborative Innovation Center, Xiamen, China; ^5^Shanghai Majorbio Bio-Pharm Biotechnology Co., Ltd., Shanghai, China

**Keywords:** *Paraphotobacterium marinum*, complete genome, two chromosomes, metabolic features, rare species

## Abstract

Members of the family *Vibrionaceae* are metabolically versatile and ubiquitous in natural environments, with extraordinary genome feature of two chromosomes. Here we reported the complete genome of *Paraphotobacterium marinum* NSCS20N07D^T^, a recently described novel genus-level species in the family *Vibrionaceae*. It contained two circular chromosomes with a size of 2,593,992 bp with G+C content of 31.2 mol%, and a plasmid with a size of 5,539 bp. The larger chromosome (Chr. I) had a genome size of 1,426,504 bp with G+C content of 31.6 mol%, and the smaller one (Chr. II) had a genome size of 1,161,949 bp with G+C content of 30.8 mol%. The two chromosomes have strikingly similar G+C contents with difference of <1% and similar percentages of coding regions. Interestingly, by comparison to 134 species affiliated with seven genera within the family *Vibrionaceae*, *P. marinum* NSCS20N07D^T^ possessed the smallest genome size and lowest G+C content. Clusters of orthologous groups of proteins functional categories revealed that the two chromosomes had different distributions of functional classes, indicating they take different cellular functions. Surprisingly, Chr. II had a large proportion of unknown genes than Chr. I. Metabolic characteristics predicted that Chr. I performed the essential metabolism, which can be complemented by the Chr. II, such as amino acids biosynthesis. Microbial community analysis of *in situ* surface seawater revealed that *P. marinum* accounted for one to four sequences among more than 20,000 of 16S ribosomal RNA gene V4 contigs, representing it apparently appeared as a rare species. What’s more, *P. marinum* was anticipated to be specific to the pelagic ocean. This study will provide new insight into more understanding the genomic and metabolic features of multiple chromosomes in prokaryote and emphasize the ecological distribution of the members in the family *Vibrionaceae* as a rare species.

## Introduction

Members of the family *Vibrionaceae* are metabolically versatile, and widely distributed in marine environments, either free-living or associated with marine animals. Interestingly, it is reported that members of the family *Vibrionaceae* have an extraordinarily ubiquitous genome feature that they possess two chromosomes (Okada et al., 2005; Dryselius et al., 2007; Dikow and Smith, 2013). The cholera pathogen *Vibrio cholerae* El Tor N16961, for example, contains two chromosomes, with the large one of 3.0 Mbp and the small one of 1.1 Mbp (Heidelberg et al., 2000). Because *V. cholerae* is the most clinically important vibrio causing cholera, a severe diarrheal disease, it becomes a model organism to study the chromosome replication initiation (Baek and Chattoraj, 2014; Gerding et al., 2015), cell division mechanisms (Galli et al., 2016), and other fundamental biological investigations. *Vibrio parahaemolyticus* KX-V237, another pathogenic vibrio causing gastroenteritis, also contains two circular chromosomes, with the large one of 3.3 Mbp and the small one of 1.9 Mbp (Tagomori et al., 2002). Although the sizes of the large chromosomes compared between the species *V.* *cholerae* and *V. parahaemolyticus* are similar, the sizes of the small ones are different (Tagomori et al., 2002). The considerably variable size of the small chromosome of the vibrios (0.8–2.4 Mbp) becomes a common genome feature of members of the family *Vibrionaceae* (Okada et al., 2005; Dryselius et al., 2007).

PacBio RS sequencing platform is called as the third-generation sequencer, which is a single-molecule, real-time sequencing technology that does not need to amplify the genomic DNA, and thus it can produce sequences with longer read length (Eid et al., 2009). By application of the PacBio RS sequencing platform, the complete and closed genome of *Burkholderia pseudomallei* with two large and complex circular chromosomes and high G+C content (68–69%) is obtained fastly and accurately (Teng et al., 2017).

We identify and describe a new genus-level species within the family *Vibrionaceae*, named *Paraphotobacterium marinum*, which is isolated from the surface seawater of the South China Sea (Huang et al., 2016). Interestingly, the genome size of *P. marinum* NSCS20N07D^T^ is 2.5 Mbp calculated from the draft genome sequence, which is much more smaller than the closely related members within the family *Vibrionaceae* (Huang et al., 2016). Here we reported the whole complete genome of *P. marinum* NSCS20N07D^T^ using PacBio RS sequencing platform, and the metabolic features were predicted with genome annotation. In addition, we performed the microbial diversity analysis of the *in situ* surface seawater where *P. marinum* NSCS20N07D^T^ was isolated, aiming to investigate the relative abundance of *P. marinum* in natural environment. Finally, we predicted the natural distribution of *P. marinum*. This study provides new insights into more understanding the genomic and metabolic features of the *Vibrionaceae* member, *Paraphotobacterium* containing two chromosomes and the ecological distribution in the ocean.

## Materials and Methods

### Genomic DNA Extraction and Quality Assessment

*Paraphotobacterium marinum* NSCS20N07D^T^ (=KCTC 52126^T^ = MCCC 1A01886^T^ = CIP 111031^T^) was cultured with Marine Broth 2216 (BD, Difco). The cell biomass of 100 ml was obtained by centrifugation at 6,000 × *g* for 10 min. The genomic DNA was extracted using the conventional phenol/chloroform/isoamyl alcohol (25:24:1) extraction method. The completeness of the extracted DNA was tested using 1.0% agarose gel electrophoresis. The concentration and purity were determined using TBS-380 and NanoDrop 2000 spectrophotometer.

### PacBio Sequencing and Sequence Assembly

A 10 kb DNA fragment library was constructed according to the manufacturer’s instruction and sequenced on the PacBio RSII sequencing platform with one SMART cell (MajorBio Co., Shanghai, China). Sequencing reads were assembled using the hierarchical genome-assembly process (HGAP 3.0) (Chin et al., 2013). A complete genome with two chromosomes and one plasmid of *P. marinum* NSCS20N07D^T^ was obtained.

### Genome Annotation

The prediction of the protein coding genes (opening reading frames, ORFs) was carried out using Glimmer 3.0 (Delcher et al., 2007). The ribosomal RNA (rRNA) genes were predicted using Barrnap^1^. The tRNA genes were predicted using tRNAscan-SE (Lowe and Eddy, 1997). Gene annotation was carried out by BLASTP search against non-redundant protein sequences (nr database), Swissprot database, the clusters of orthologous groups of proteins (COG) database, and KEGG database (Kanehisa et al., 2016) with *e*-value cutoff of 1e-5 (Tatusov et al., 2001). Further annotation was performed using the RAST server online (Aziz et al., 2008) and MicroScope platform (Vallenet et al., 2009). The circular genome was drawn using Circos (Krzywinski et al., 2009).

### Microbial Composition of the *in Situ* Surface Seawater and Relative Abundance of *P. marinum*

The *in situ* surface seawater of two sites (NSCS1 [119°26′24.62′′E, 21°58′35.71′′N] and NSCS2 [117°36′11.76′′E, 19°19′17.30′′N]) in the South China Sea was collected on May 14–15, 2015. *P. marinum* NSCS20N07D^T^ was isolated from site NSCS2 at a depth of 3,048 m (Huang et al., 2016). Triplicate 2 l seawater of each sampling site was filtered on board using 0.22 μm filter membrane (GF, Millipore, United States). The membrane was put into a 2.0 ml EP tube and store at -20°C until use.

The total DNA of filtered surface seawater was extracted using the traditional proteinase K/SDS lysis followed by phenol/chloroform/isoamyl alcohol (25:24:1) extraction method (Huang et al., 2014). The quality of total DNA was evaluated by 1.0% agarose gel electrophoresis. The 16S rRNA gene V4 region was amplified with the specific primers 515F (GTGCCAGCMGCCGCGGTAA) and 806R (GGACTACHVGGGTWTCTAAT) with dual barcode (Kozich et al., 2013). The PCR amplification mixture was made in 50 μl reactions with total DNA of 10 ng. The PCR reaction was conducted with TransStart Fastpfu DNA Polymerase as follows: initial denaturation at 98°C for 1 min, followed by 30 cycles of denaturation at 98°C for 10 s, annealing at 55°C for 30 s and elongation at 72°C for 30 s, and extension at 72°C for 5 min. For each sample, three parallel PCR reactions were carried out and pooled together before purification of the PCR product with AxyPrepDNA gel extraction Kit (Axygen). The high-throughput sequencing of the 16S rRNA gene V4 region was performed using Illumina Miseq PE250 platform (Illumina, San Diego, CA, United States).

The pair-end reads of each sample were merged into contigs using make.contigs command implemented in mothur v.1.34.4 (Schloss et al., 2009). The contigs were then trimmed using Qiime pipeline (Caporaso et al., 2010) as follows: (1) allowing no mismatch with dual barcode and primers; (2) allowing no ambiguous bases; and (3) removal of contigs containing the homopolymers over 6. Then, the chimeras were checked using usearch61 algorithm against the latest Greengenes database of gg_13_8_97_otus (DeSantis et al., 2006). The operation taxonomic unit (OTU) was picked with a 97% similarity threshold using the *de novo* picking method.

The contigs of V4 region of each sample affiliated with *P. marinum* were identified by mapping to the full-length 16S rRNA gene of *P. marinum* NSCS20N07D^T^ (1,539 bp, accession number: KU140414) using BLASTN with *e*-value of 1*e*-5 and sequence similarity of >97%.

### Ecological Distribution of *P. marinum*

The closely related sequences of *P. marinum* were searched in GenBank using Nucleotide BLAST using the full-length 16S rRNA gene of *P. marinum* NSCS20N07D^T^. The sequence similarity above 97% with *P. marinum* NSCS20N07D^T^ was determined as the same species, and the isolation source of the closely related sequences was summarized.

## Results and Discussion

### Genome Features of *P. marinum* NSCS20N07D^T^

**FIGURE 1:**
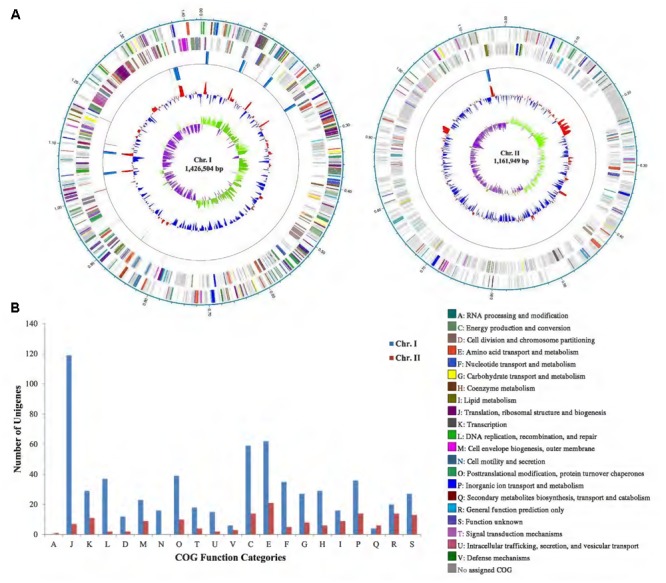
**(A)** Circular genome maps of *P. marinum* NSCS20N07D^T^ containing two chromosomes. From the inside to outside: GC-skew; GC content; *rrn* operon; and tRNA genes; ORFs on the reverse strand (colorized by COG functional categories); ORFs on the forward strand (colorized by COG functional categories); locations in megabases (Mb). **(B)** COG functional categories of annotated ORFs in Chr. I and Chr. II of *P. marinum* NSCS20N07D^T^. COG included three large groups: information storage and processing (A, J, K, L); cellular processes and signaling (D, M, N, O, T, U, V), metabolism (C, E, F, G, H, I, P, Q), and poorly characterized (R, S).

A total of 131,203 clean reads were obtained with the largest of 40,999 bp retrieved from one SMART cell of PacBio RSII sequencing. The complete genome of *P. marinum* NSCS20N07D^T^ was obtained, containing two circular chromosomes with a total size of 2,593,992 bp with G+C content of 31.2 mol%, and one plasmid with a size of 5,539 bp. The larger chromosome (Chr. I) had a genome size of 1,426,504 bp with G+C content of 31.6 mol%, and the smaller one (Chr. II) had a genome size of 1,161,949 bp with G+C content of 30.8 mol% (**Figure 1** and **Table 1**).

**Table 1:** Comparison of genome features of *P. marinum* with other representative species in the family *Vibrionaceae* containing two circular chromosomes.

Species	1		2		3		4		5		6		7		8	
Chromosomes (Chr.)	Chr. I	Chr. II	Chr. I	Chr. II	Chr. I	Chr. II	Chr. I	Chr. II	Chr. I	Chr. II	Chr. I	Chr. II	Chr. I	Chr. II	Chr. I	Chr. II
Size (bp)	1,426,504	1,161,949	4,085,304	2,237,943	3,856,884	2,052,529	2,897,536	1,330,333	3,325,165	1,206,461	2,961,149	1,072,315	3,288,558	1,877,212	3,215,193	783,080
GC content (mol%)	31.6	30.8	42.0	41.2	50.7	47.9	39.0	37.0	39.2	38.2	47.7	46.9	45.4	45.4	49.7	48.6
ORFs (Protein)	1,481	1,148	3,383	1,921	3,284	1,695	2,584	1,174	2,901	1,017	2,534	970	3,079	1,752	2,690	678
*rrn* operons	8	1	14	1	9	0	11	1	11	1	8	0	10	1	9	0
5S rRNA^∗^	1	0	2	1	2	0	1	0	1	0	1	0	1	0	1	0
tRNAs	69	7	150	19	116	15	107	11	91	13	94	4	112	14	104	1

1, *P. marinum NSCS20N07D^T^; 2, *Photobacterium profundum* SS9 (NC_006370, NC_006371); 3. *Photobacterium gaetbulicola* Gung47 (NZ_CP005974, NZ_CP005973); 4, *Aliivibrio fischeri* ES114 (NC_006840.2, NC_006841.2); 5, *Aliivibrio salmonicida* LFI1238 (NC_011312.1, NC_011313.1); 6, *Vibrio cholerae* O1 biovar El Tor str. N16961 (NC_002505.1, NC_002506.1); 7, *Vibrio parahaemolyticus* RIMD 2210633 (NC_004603.1, NC_004605.1); 8, *Grimontia hollisae* ATCC 33564 (NZ_CP014056.1, NZ_CP014055.1). *rrn* operons represent the number of 16S–23S–5S rRNA. ^∗^Extra copies. Data of two to eight were obtained from the genome database in GenBank*.

**FIGURE 2:**
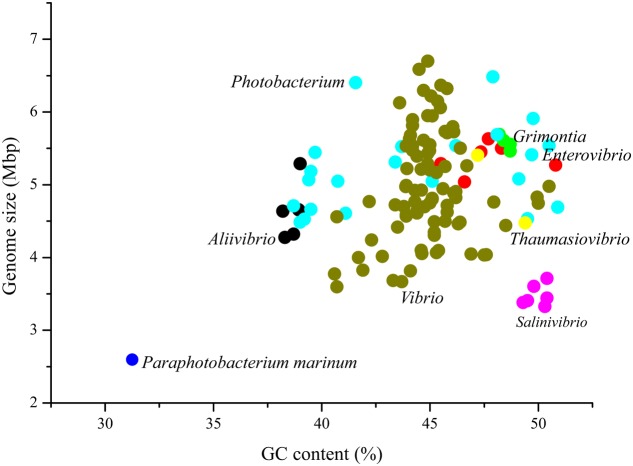
Genome size and GC content of *P. marinum* NSCS20N07D^T^ in comparison with other species in the family *Vibrionaceae*. There were 134 genomes available in the genome resource in NCBI database, belonging to the following seven genera, *Aliivibrio* (5), *Enterovibrio* (6), *Grimontia* (5), *Photobacterium* (22), *Salinivibrio* (6), *Thaumasiovibrio* (2), and *Vibrio* (88). The number in bracket indicated the number of genomes within each genus. Although there were two genomes smaller than *P. marinum* NSCS20N07D^T^, “*Candidatus* Photodesmus blepharushas” of 1.11231 Mbp with GC content of 35.6%, and “*Candidatus* Photodesmus katoptron” of 1.01592 Mbp with GC content of 30.9%, they were not described as a species, since they were organism-specific and uncultured yet. Thus, the two genomes were excluded in this study.

By comparison to 134 species affiliated with seven genera in the family *Vibrionaceae*, *P. marinum* NSCS20N07D^T^ possessed the smallest genome size and lowest G+C content (**Figure 2**). As far as we know, *P. marinum* NSCS20N07D^T^ was the smallest genome in *Vibrionaceae* species reported so far.

To compare *P. marinum* with other *Vibrionaceae* species in more details on each chromosomes, we selected seven representative species with complete genome, *Photobacterium profundum* SS9, *Photobacterium gaetbulicola* Gung47, *Aliivibrio fischeri* ES114, *Aliivibrio salmonicida* LFI1238, *V. cholerae* O1 biovar El Tor str. N16961, *V. parahaemolyticus* RIMD 2210633, and *Grimontia hollisae* ATCC 33564 (**Table 1**). Obviously, Chr. I of *P. marinum* NSCS20N07D^T^ was the smallest chromosome among those of the above representative species, the size of which ranged from 2,897,536 bp of *A. fischeri* ES114 to 4,085,304 bp of *P. profundum* SS9. Strangely, Chr. II of *P. marinum* NSCS20N07D^T^ was not the smallest. The size of the smaller chromosome showed considerably variable pattern among the compared members of the family *Vibrionaceae*, ranging from 783,080 to 2,237,943 bp (**Table 1**). The variable size of Chr. II was recognized as more flexible genes therein (Okada et al., 2005). G+C content of the two circular chromosomes of *P. marinum* NSCS20N07D^T^ had less than 1% difference. This was in congruence with the genome characteristics of *Vibrionaceae* species, which was regarded as a long evolutionary co-existence for the divided chromosomes (Okada et al., 2005). Furthermore, on the aspect of genome size, the difference between Chr. I and Chr. II was much smaller in *P. marinum* NSCS20N07D^T^ than the corresponding other species within this family (**Table 1**).

Through gene annotation, we found that Chr. I encoded 1,481 ORFs, and had 8 copies of *rrn* operon (16S–23S–5S rRNA genes), an extra 5S rRNA gene, and 69 tRNA genes; while Chr. II encoded 1,148 ORFs and had 1 copy of *rrn* operon and 7 tRNAs. It was noteworthy that all these 16S rRNA genes shared 100% sequence identity (**Figure 1** and **Table 1**).

By means of BLASTP search against nr database, Chr. II had a larger proportion of unknown proteins and hypothetical proteins, accounting for 29.4 and 29.8%, respectively, indicating there was a larger proportion of genes not yet elucidated (**Supplementary Table S1**).

### COG Categories

By COG functional annotating, we found that 42.47% in Chr. I and 13.50% in Chr. II can be annotated. Surprisingly, a huge amount of ORFs (86.50%) in Chr. II could not be classified (No assigned COG in **Figure 1**). This was in congruence with the result of BLASTP against nr database, indicating that the biological function of most genes in Chr. II was still unknown. On the other hand, the vast majority of genes for essential cell function were located in Chr. I determined from COG classes, such as Class J of translation, ribosomal structure, and biogenesis; Class C of energy production and conversion; Classes of D, E, F, G, and P of transport; and Class L of replication, recombination, and repair. Cell motility and secretion (Class N) were undertaken mainly by Chr. I other than Chr. II. COG functional categories revealed that the two chromosomes have different distributions of functional classes, indicating they take different cellular functions.

### Replication Initiation

The study on chromosome replication of the two chromosomes of *V. cholerae* attracted much more attention in recent years (Baek and Chattoraj, 2014; Val et al., 2016). *DnaA* was reported as the main controller in Chr. I in *V. cholerae* (Baek and Chattoraj, 2014). Similarly, in *P. marinum* NSCS20N07D^T^, the replication initiation region of Chr. I was identified by the gene configuration of *dnaA*, *dnaN*, and *recF*, which encoded chromosomal replication initiation protein DnaA, DNA polymerase III subunit beta, and DNA replication/repair protein RecF, respectively (**Supplementary Table S1**). CrtS, the RctB binding locus, was reported to mediate the replication initiation of Chr. II (Baek and Chattoraj, 2014). However, in *P. marinum*, we did not find CrtS, whereas the replication initiation region of Chr. II was identified by chromosome partitioning protein ParB (orf00002_1) and protein ParA (orf00003_1). With respect to the plasmid replication, there was a RepA protein found in the plasmid of *P. marinum*, which can control the replication of plasmid.

### Carbohydrate Metabolism

**FIGURE 3:**
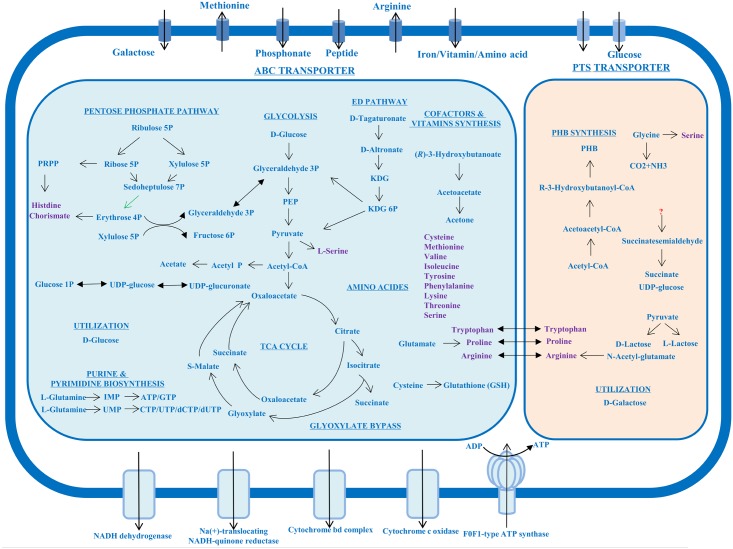
Overview of metabolic characteristics of *P. marinum* NSCS20N07D^T^. The large blue box and the small orange box showed the metabolic pathway in Chr. I and Chr. II, respectively. Amino acids were marked in purple. The double sided arrows across the two boxes represented these amino acids synthesis genes were encoded in the two chromosomes. The green arrow represented the gene involved in this step was encoded in Chr. II. KDG, 2-dehydro-3-deoxy-D-gluconate; PEP, phosphoenolpyruvate; PHB, poly-β-hydroxybutyrate.

Complete genes taking part in the central carbohydrate metabolism could be found in Chr. I (**Figure 3**), including the Embden–Meyerhof–Parnas pathway (EMP or glycolysis), pyruvate oxidation to acetyl-CoA, tricarboxylic acid (TCA) cycle, and glyoxylate bypass (also called glyoxylate cycle). Chr. I also contained an Entner–Doudoroff pathway, which could replace the glycolysis to catabolize glucose to pyruvate. Most genes involved in pentose phosphate pathway were encoded in Chr. I, however, the transaldolase catalyzing the step of sedoheptulose 7-phosphate and glyceraldehyde 3-phosphate to form fructose 6-phosphate and erythrose 4-phosphate was encoded in Chr. II. This indicated the pentose metabolism could be regulated by the smaller chromosome Chr. II.

On the aspect of utilization of carbon source, the two chromosomes hosted different genes encoding different metabolic activities. Chr. I possessed the genes encoding the utilization of D-glucose, while Chr. II mainly possessed those for the utilization of D-galactose. This demonstrated that Chr. II improved the ability of the metabolic range of carbon source. No data supported that this bacterium can utilize oligosaccharides.

### Amino Acids Biosynthesis and tRNAs

Through metabolic pathway analysis, 13 kinds of amino acids can be synthesized, which were found in Chr. I. The majority of genes involved in arginine biosynthesis could be found in Chr. II except the gene *argD* encoding acetylornithine aminotransferase, which was encoded in Chr. I (**Supplementary Table S1**). Tryptophan and proline biosynthesis were also annotated in the two chromosomes (**Figure 3**).

Through gene annotation, Chr. I contained 19 kinds of tRNA synthetase responsible for the 19 amino acids except proline. Chr. II contained only two tRNA synthetases for proline and tryptophan, respectively.

### Poly-β-Hydroxybutyrate Biosynthesis and Degrading Pathway

Strain NSCS20N07D^T^ can produce a large amount of poly-β-hydroxybutyrate (PHB) granules inside the cell observed using transmission electron microscopy (TEM) after negative staining and Nile blue A stain (Huang et al., 2016). Gene annotation and metabolic analysis revealed that a gene cluster (*atoB*, *phbB*, and *phbC*) was present in Chr. II for PHB biosynthesis from two acetyl-CoA molecules to form acetoacetyl-CoA (**Figure 3**), whereas it seems that the degrading of PHB was controlled by Chr. I as the incomplete pathway from (*R*)-3-hydroxybutanoate to acetone was found, but the mechanism of polymer hydrolyze needed further study.

### Flagella

All genes encoding for flagellar biosynthesis proteins could be found in Chr. I (**Supplementary Table S1**), indicating that strain NSCS20N07D^T^ could be motile. However, no flagella were observed by TEM after negative stain (Huang et al., 2016), making it likely that the flagella were lost in sample preparation.

### Bacterial Relative Abundance of *P. marinum* in *in Situ* Surface Seawater

**FIGURE 4:**
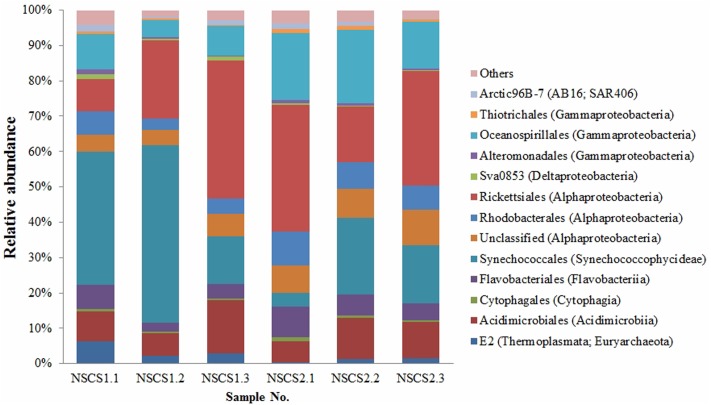
Relative abundance of microbial community of the surface seawater collected from the South China Sea.

To investigate the relative abundance of *P. marinum* in the surface seawater where it was isolated, we carried out microbial diversity analysis based on high-throughput sequencing of 16S rRNA gene V4 region using Miseq platform. The six samples produced a total of 142,564 contigs with the minimum of 18,669 contigs for NSCS1.3 and the maximum of 28,067 contigs for NSCS1.1. The surface seawater of the sampling sites in South China Sea was dominated by *Cyanobacteria*, *Proteobacteria*, *Bacteroidetes*, and *Actinobacteria*, though the data seemed to be variable among each samples (**Figure 4**). *Synechococcales* in *Cyanobacteria* (3.82–50.19%), *Rickettsiales* in *Alphaproteobacteria* (9.10–38.90%), *Oceanospirillales* in *Gammaproteobacteria* (4.97–20.89%), and *Acidimicrobiales* in *Actinobacteria* (6.11–15.05%) were the dominant groups in the surface seawater (**Figure 4**). *Flavobacteriales* of the class *Flavobacteriia* accounted for 2.47–8.74% relative abundance (**Figure 4**). While sequences affiliated with the family *Vibrionaceae* accounted for less than 0.1% of relative abundance.

By mapping the V4 contigs to the full-length 16S rRNA gene of *P. marinum* NSCS20N07D^T^, we found that there were four contigs (253 bp in length) in NSCS1.1 (28,067 contigs), one contig in each sample of NSCS1.2 (25,062 contigs), NSCS2.1 (24,463 contigs), and NSCS2.2 (22,064 contigs), having 99.6–100% similarity with the 16S rRNA gene of *P. marinum*, while other 16S rRNA gene V4 contigs showed the similarity no more than 96.0%. The seven sequences were classified into a single OTU (denovo_289). The low relative abundance of *P. marinum* indicated that it was a rare bacterial species which were regarded as the threshold of five sequences per OTU (Skopina et al., 2016). Thus, it is assumed that *P. marinum* is present as a rare species in the surface seawater.

### Ecological Distribution of *P. marinum*

Through 16S rRNA gene similarity search against the Nucleotide collection (nr/nt) database, we found that this bacterium *P. marinum* showed 99% similarity with the 21 uncultured bacterium clones detected from the Red Sea coral *Pocillopora damicornis* (accession number: KC668602), seawater from South China Sea (GU061990, GU061981), hydrothermal plumes of East Pacific Rise (JN874365, JN874376, JN874374, JN873939, JN874354, JN874093, JN874308, JN874318, JN874322), West Pacific (KF271041), and Gulf of Mexico oil sheen (KF786540, KF786512, KF786461), ocean water of north eastern Gulf of Mexico (KU578418), and CALCOFI Line 67-155 depth of 5 m size fraction 3.0–20 μm (KX935264, KX935265, KX935266, KX935267). These environments, to some extent, are all pelagic niches that are distinct from the coast region. Thus, we speculate that *P. marinum* is specific to the pelagic environment, and has a global distribution by free-living, or coral-associated, and from surface to the deep extreme hydrothermal environments.

## Conclusion

In conclusion, this is the first report of the complete genome sequence and metabolic features of *P. marinum* containing two chromosomes. By comparison to other members in the family *Vibrionaceae* on the aspect of genome features, *P. marinum* has the smallest genome reported so far. Metabolic characteristics predict that Chr. I and Chr. II have different genes, indicating they perform different cellular functions. A large proportion of unknown genes is present in the small chromosome, highlighting the main functions of Chr. II remain unclear. Finally, *P. marinum* is assumed to be specific to pelagic environment, and widely distributed in the global ocean as a rare species. Our study will provide new insights into further understanding the genomic features of *Vibrionaceae* members containing two chromosomes, and the multipartite metabolic features of multiple chromosomes in prokaryote, and emphasize the ecological distribution of *Vibrionaceae* species as a rare species.

## Nucleotide Sequence Accession Numbers

The whole complete genome sequence of *P. marinum* NSCS20N07D^T^ has been deposited in GenBank under accession number CP022355-CP022357.

## Author Contributions

ZH and ZS conceived this study. ZH performed the data analysis. ZL conducted sequence assembly and genome annotation. ZH and ZS wrote the manuscript.

## Conflict of Interest Statement

The authors declare that the research was conducted in the absence of any commercial or financial relationships that could be construed as a potential conflict of interest.

## Footnotes

1 http://www.vicbioinformatics.com/software.barrnap.shtml

## Supplementary Material

TABLE S1 Genome annotation of the two chromosomes of *P. marinum* NSCS20N07D^T^. Chr. I and Chr. II represent the larger and smaller chromosomes, respectively. The genes are grouped together according to the metabolic pathway.
